# Oral health status among 60-year-old individuals born in 1941–1943 and 1954–1955 and 81-year-old individuals born in 1922–1924 and 1933–1934, respectively: a cross-sectional study

**DOI:** 10.1007/s00784-022-04632-5

**Published:** 2022-07-29

**Authors:** Sladjana Critén, Pia Andersson, Stefan Renvert, Bengt Götrick, Johan Sanmartin Berglund, Viveca Wallin Bengtsson

**Affiliations:** 1 Faculty of Health Sciences, Kristianstad University, 291 88 Kristianstad, Sweden; 2 Department of Health, Blekinge Institute of Technology, 371 79 Karlskrona, Sweden; 3 Faculty of Odontology, Malmö University, 205 06 Malmö, Sweden

**Keywords:** Aging, Epidemiology, Older, Oral health status

## Abstract

**Objective:**

This study aimed to analyze the oral health status of four different birth cohorts: two cohorts of 60-year-olds born in 1941–1943 and 1954–1955 and 2 cohorts of 81-year-olds born in 1920–1922 and 1933–1934.

**Material and methods:**

The study was based on data from an ongoing longitudinal population project, The Swedish National Study on Aging and Care (SNAC). Oral health status was repeatedly examined clinically and radiographically in 2001–2003 and 2014–2015, including 60- and 81-year-olds, in total 412 individuals. Statistical analyses were performed using independent-samples *t* test and Pearson’s χ^2^ test.

**Results:**

More individuals were dentate in 2014–2015 compared to 2001–2003 in the two age groups: 60 and 81 years (*p* < 0.001 for both). The mean number of teeth increased in the 60-year-olds from 24.2 to 27.0 and in the 81-year-olds from 14.3 to 20.2. The numbers of at least one intact tooth increased for both age groups (*p* < 0.001 and *p* < 0.004, respectively). In the age groups 81 years, there was an increase in having at least one PPD ≥ 6 mm (*p* < 0.016) and bone loss ≥ 5 mm (*p* < 0.029) between the two examinations. No such differences were found in the age groups of 60 years.

**Conclusion:**

Over 13 years, oral health improved for both 60- and 81-year-old age groups. The most significant changes were in the 81-year-olds where oral health had improved except for periodontal status.

**Clinical relevance:**

More natural teeth and impaired periodontal status potentially impact oral health and should increase focus on preventive and supportive dental care in older individuals.

## Introduction

The older population is worldwide steadily growing [1]. The proportion of individuals 60 years and older is expected to increase from 12 to 22% between 2015 and 2050 [2]. In Sweden, 20% of the population was 65 years or older in 2020 [3]. There is an increased risk that older individuals will suffer from illnesses and injuries [4, 5]. Cellular function appears to decrease with increasing age which may cause risks for chronical diseases and frailty [6]. Increased prevalence of diseases leads to increased use of medication. Both illness and medications can negatively affect oral health due to decreased salivary flow that can lead to dry mouth and modified saliva composition [7–9]. These side effects can lead to chewing and swallowing difficulties [10, 11]. In addition, oral pathogenic bacterial community may increase which can trigger host response and increase risks for developing of oral diseases [12]. Diseases and disabilities can lead to a reduced ability for older individuals to manage oral hygiene self-care [13]. Difficulties in performing adequate oral hygiene increase the risk of developing oral diseases [14, 15].

Oral diseases have been stated as number four of global issues [16]. It has been reported that advanced periodontitis and untreated caries lesions have globally increased by 40% between 1990 and 2015, and these diseases are the primary reason for tooth loss. In 2015, the state of untreated caries in permanent teeth was still the most prevalent oral condition worldwide affecting around 34% of the adult population [17]. The prevalence of advanced periodontitis affects between 9 and 11% of the world’s adult population [18, 19]. Both caries and periodontal diseases tend to increase after 60 years [20] Epidemiological studies have shown that periodontitis increases with increasing age [21, 22] and affects more frequently men [22, 23]. Untreated caries lesions and periodontitis may incorporate pain, abscesses, and tooth extractions and lead to impaired quality of life [17]. In addition, pain in the mouth may limit social life [24].

Generally, the dentition, periodontium, oral and masticatory mucosa, and salivary function undergo natural changes with increasing age. These changes can disable the oral health status functionally which combined with increased bacterial presence and underlying medical issues can lead to development and progression of oral diseases [25]. Therefore, more older individuals are a significant challenge for dental care and other healthcare actors [26]. Oral health is an essential part of healthy aging, and poor oral health status can adversely affect general health [5] and increase risks for dependency of care [20]. The number of missing and/or carious teeth is associated with cardiovascular disease, diabetes, male gender, and age (82–91 years) [27]. Periodontitis can be a risk factor for developing heart diseases and death [28]. To prevent oral diseases and maintain a good oral health, treatment planning in older individuals with both preventive and supportive regular care must be considered [29]. This can be cost-effective for both individuals and society [30]. Thus, it is crucial to seek awareness of aging and oral health status among older individuals to improve dental care and oral health interventions.

This study aimed to analyze the oral health status of four different birth cohorts: two cohorts of 60-year-olds born in 1941–1943 and 1954–1955 and 2 cohorts of 81-year-olds born in 1920–1922 and 1933–1934.

## Materials and methods

### Study population

The study was conducted as a cross-sectional study, using quantitative methods. It was based on data from the ongoing longitudinal population-based project The Swedish National Study on Aging and Care (SNAC) in Karlskrona, in southeastern Sweden. The SNAC study started in 2001. Participants in the study were randomly selected from a national population database and included urban and rural areas from the municipality of Karlskrona and individuals aged 60–96 years. An equal randomly selection among individuals in age groups 60, 66, 72, and 78 were made, whilst all individuals in age groups 81, 84, 87, and older were invited to participate. All individuals were invited by regular mail. The proportion of enrolled individuals was 62% corresponding to 10% of Karlskrona’s population. The data collection is ongoing, and every sixth year, a new cohort of 60 years and 81 years of age are invited to participate.

During the years 2001–2003 (exam 1), 263 individuals aged 60 years born in 1941–1943 were invited, and 191 chose to participate, while in 2014–2015 (exam 2), born between 1954 and 1955, 116 individuals were invited, and 69 decided to participate. Of individuals aged 81 years at exam 1, 254 born 1922–1924 were invited, and 155 individuals chose to participate. At exam 2, 119 individuals born 1933–1934 were invited to participate, and 68 decided to participate. All study participants signed informed consent before the start of the study. The participants who could not receive an oral examination at the dental clinic were offered to be examined in their own home. The study procedure and study population details have been described in other studies [31, 32].

Inclusion criteria in the present study were all participants who underwent an oral health examination at the dental clinic including a panoramic radiograph. Additional inclusion criteria were that individuals should be residents in Karlskrona municipality and be aged 60 years or 81 years at exams 1 and 2. In total, 412 individuals met the inclusion criteria.

The ethical rules described in the Helsinki Declaration [33] were followed. The Research Ethics Committee has approved ethical permission for the SNAC studies at the Lund University, Lund, Sweden (LU 604–00).

### Oral examination

All included participants underwent an oral health examination, including a panoramic radiograph (Orthopantomograph OP 100, Instrumentarium, Tuusula Finland) by two experienced dental hygienists who were calibrated. At exam 1, the panoramic radiograph technique was analogue, whereas it was digital at exam 2. When conducting the clinical examination, a structured protocol was used. The oral health examination has been carried out at a dental clinic.

The panoramic x-ray images and the x-ray data were coded and assessed by two calibrated dentists, one at each period. Calibration between dentist 1 in 2001 and dentist 2 in 2014 was done by randomizing 10% of panoramic x-ray from 2001, on which dentist 2 calibrated. The clinical variables, including third molars, were number of teeth, number of intact teeth, dental implants, presence of removable prostheses, buccal/lingual caries, pocket depth (PPD), bleeding on probing (BOP), and mucosal-plaque index (MPI). In addition, the number of teeth, dental implants, root residues, root fillings, approximal caries, and bone loss was assessed from radiographs. A revaluation was done if there was uncertainty about any of these variables.

The occurrence of manifest caries was registered clinically by visual and tactile examination of buccal and lingual surfaces using a mouth mirror, a blaster, and a double-ended EXD 5 probe, (Hu-Friedy Inc. Chicago, IL). In addition, the presence of manifest approximal caries was registered by using a panoramic radiograph. The ICC correlation coefficient between the two observer measurements on panoramic radiographs was 0.96 (95% CI 0.88 to 0.98) and was based on total of 20 observations.

The criteria for bacterial coatings on the teeth the plaque score were calculated using the mucosal plaque index (MPI index) [34]. The index is based on visual grading from 1 to 4: no soft coatings (grade I), small amounts of barely visible coatings (grade II), moderate amounts of coatings (grade III), and ample amounts of cohesive coatings (grade IV).

Probing pocket depth (PPD) and bleeding on probing (BOP) on four surfaces around each tooth were performed using a CP-12 probe (Hu-Friedy Inc. Chicago, IL). The deepest pocket depth from 4 mm was registered. Therefore, only PPD ≥ 5 mm are presented in this study. BOP was registered as bleeding or not on all surfaces and was calculated as the proportion of teeth with bleeding. Gingivitis was classified if BOP was ≥ 10%. Bone loss was measured by panoramic radiograph as the extent of alveolar bone loss from the cement enamel junction (CEJ) to the highest marginal bone level on the mesial and distal surface of each tooth.

Reliability measurements between randomly selected cases for double assessments regarding the inter-observer agreement of PPD values between the two clinicians performing the examinations were 0.76 (Cronbach’s α) (95% CI 0.67 to 0.82). The ICC correlation coefficient between the two observer measurements was 0.86 (95% CI 0.65 to 0.94) and was based on total of 20 observations, concerning the current number of approximal surfaces with 5 mm distances CEJ-marginal bone on panoramic radiograph. A diagnosis of periodontitis was declared if BOP ≥ 10% + PPD ≥ 5 mm at ≥ 2 surfaces + bone loss ≥ 5 mm.

### Statistical analyses

The Statistical Package for the Social Science (IBM SPSS, version 24.0) was used for descriptive and analytical statistics. Descriptive statistics including means, standard deviation (SD), and frequency distribution were summarized concerning age groups and examination based on time intervals for exam 1 and exam 2. Independent-samples *t* test was conducted to analyze differences between the cohorts (equal variance not assumed). Dichotomous data were analyzed using Pearson’s χ^2^ test. Statistical significance was determined at *p* < 0.05.

## Results

In the age cohorts 60 years at exam 1, 172 individuals (51% women) participated. The corresponding figure at exam 2 was 64 individuals (53% women). The age cohorts 81 years had 125 (51% women) individuals at exam 1 and 51 (43% women) at exam 2. In Table 1, the reasons for internal dropout leading to exclusion are reported.

**Table 1:** An overview of reasons to exclusion in the cohorts aged 60 years and 81 years at exam 1 and at exam 2 in relation to gender

Reasons for exclusion	Exam 1 60 y *n* = 191 *n* (%)	Exam 2 60 y *n* = 69 *n* (%)	Exam 1 81 y *n* = 155 *n* (%)	Exam 2 81 y *n* = 68 *n* (%)
Lack of panoramic radiograph				
Women	2 (1.0)	1 (1.4)	-	1 (1.5)
Men	1 (0.5)	-	-	1 (1.5)
Could not get to the dental clinic				
Women	-	-	6 (3.9)	6 (8.8)
Men	-	-	3 (1.9)	5 (7.3)
Declined the dental examination				
Women	7 (3.7)	1 (1.4)	14 (20.6)	1 (1.5)
Men	10 (5.2)	-	7 (10.3)	-
Most of survey is missing				
Women	-	2 (2.9)	-	2 (2.9)
Men	-	1 (1.4)	-	1 (1.5)

### *Cohort groups: 60 years*

**Fig. 1:**
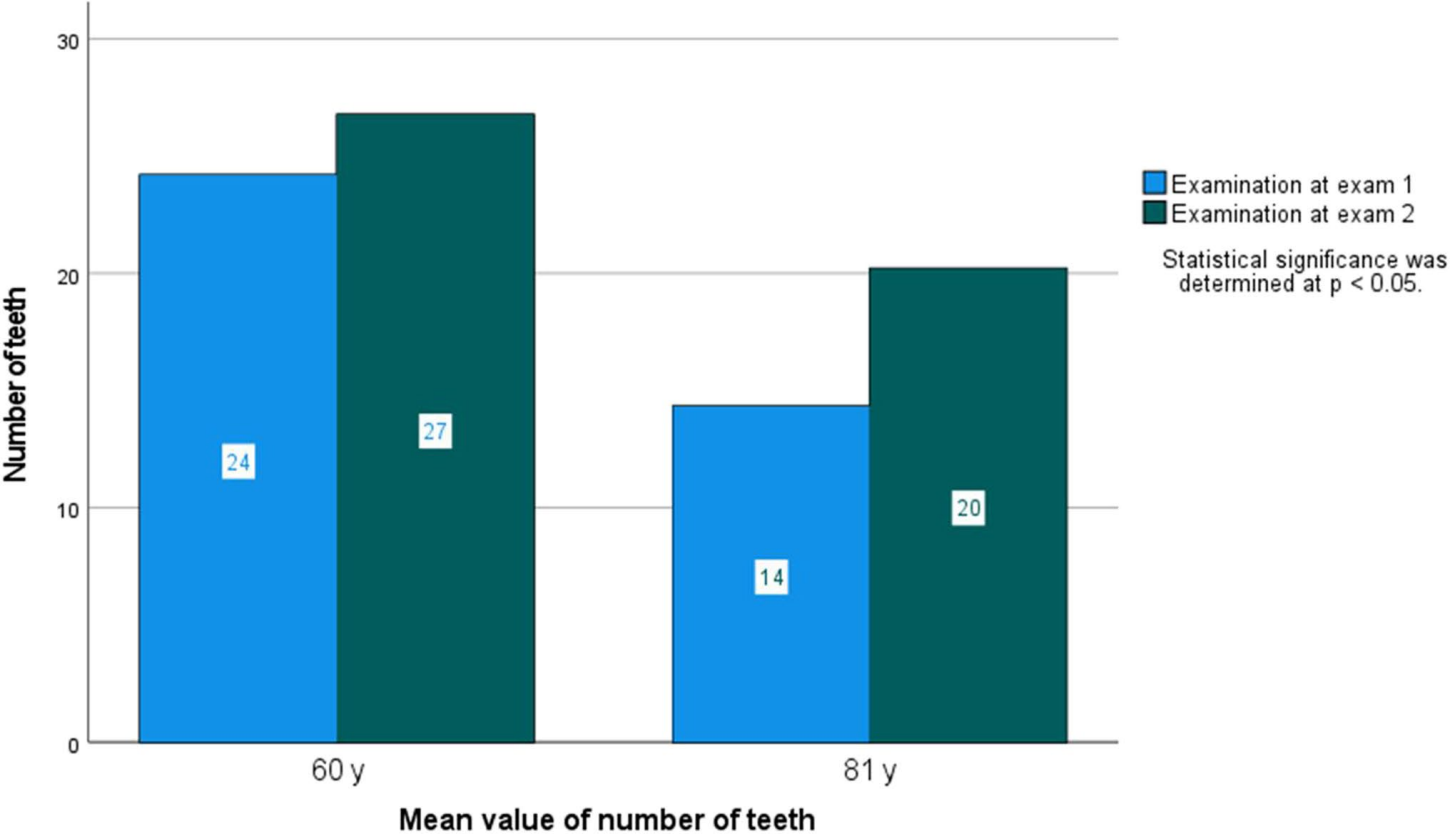
Mean value of number of teeth in the age cohorts 60 years and 81 years examined at exam 1 and exam 2

The percentage of the dentate individuals increased from 97.0% at exam 1 to 100% at exam 2. The mean number of teeth increased from 24.2 (SD ± 5.07) at exam 1 to 27.0 (SD ± 3.39) at exam 2 (*p* < 0.001) (Fig. 1), and the presence of 20 teeth or more among the individuals increased from 85.8% at exam 1 to 96.8 at exam 2 (*p* < 0.017).

Removable prostheses were found in 5.5% at exam 1, while no individuals at exam 2 had removable prostheses. Dental implants were present in 3% at exam 1 (mean 5.0, SD ± 2.24) and in 1.6% at exam 2 (mean 2.0, SD ± 0) (NS).

The mean number of intact teeth in all dentate individuals, root residues, root fillings, and manifest buccal/lingual and approximal caries with respective conditions is presented in Table 2. There were no significant differences in the prevalence of intact teeth, root residues, and approximal caries except for root fillings and buccal/lingual caries. The proportion of individuals with one or more root fillings was higher at exam 1 (85.2%) compared to exam 2 (53.1%) (*p* < 0.001). Fewer individuals had buccal/lingual caries at exam 1 (5.7%) compared to exam 2 (17.7%) (*p* < 0.008).

**Table 2:** Mean number of intact teeth, root residues, root fillings, and manifest buccal/lingual and approximal caries, respectively, in dentate individuals of age cohorts 60 years and 81 years at exam 1 and at exam 2

Variables	Exam 1 60 y *n* = 165	Exam 2 60 y *n* = 64	*p* value	Exam 1 81 y *n* = 101	Exam 2 81 y *n* = 48	*p* value
Intact teeth						
Mean (SD)	5.21 (4.14)	9.56 (6.02)	0.001^§^	2.73 (3.17)	4.65 (3.84)	0.004^§^
Range	0–18	0–27		0–15	0–13	
Total, *n*	164	64		99	48	
Root residues						
Mean (SD)	1.40 (0.55)	2.20 (1.79)	NS	1.56 (0.73)	1.33 (0.52)	NS
Range	1–2	1–5		1–3	1–2	
Total, *n*	5	5		9	6	
Root fillings						
Mean (SD)	3.45 (2.14)	2.24 (1.76)	0.003^§^	3.71 (2.37)	3.51 (2.15)	NS
Range	1–13	1–9		1–13	1–9	
Total, *n*	138	34		90	39	
Teeth with buccal/lingual caries						
Mean (SD)	1.78 (1.20)	1.82 (1.54)	NS	1.50 (0.93)	1.14 (0.38)	NS
Range	1–4	1–6		1–3	1–2	
Total, *n*	9	11		8	7	
Teeth with approximal caries						
Mean (SD)	1.44 (0.63)	1.44 (0.73)	NS	1.42 (0.88)	1.33 (0.65)	NS
Range	1–3	1–3		1–5	1–3	
Total, *n*	16	9		24	12	

Notes: Statistical analysis by independent *t* test (equal variance not assumed) was used; Mean intact teeth was calculated on all teeth in dentate individuals; ^§^Statistical significance was determined at *p* < 0.05; *NS*, non-significant

**Fig. 2:**
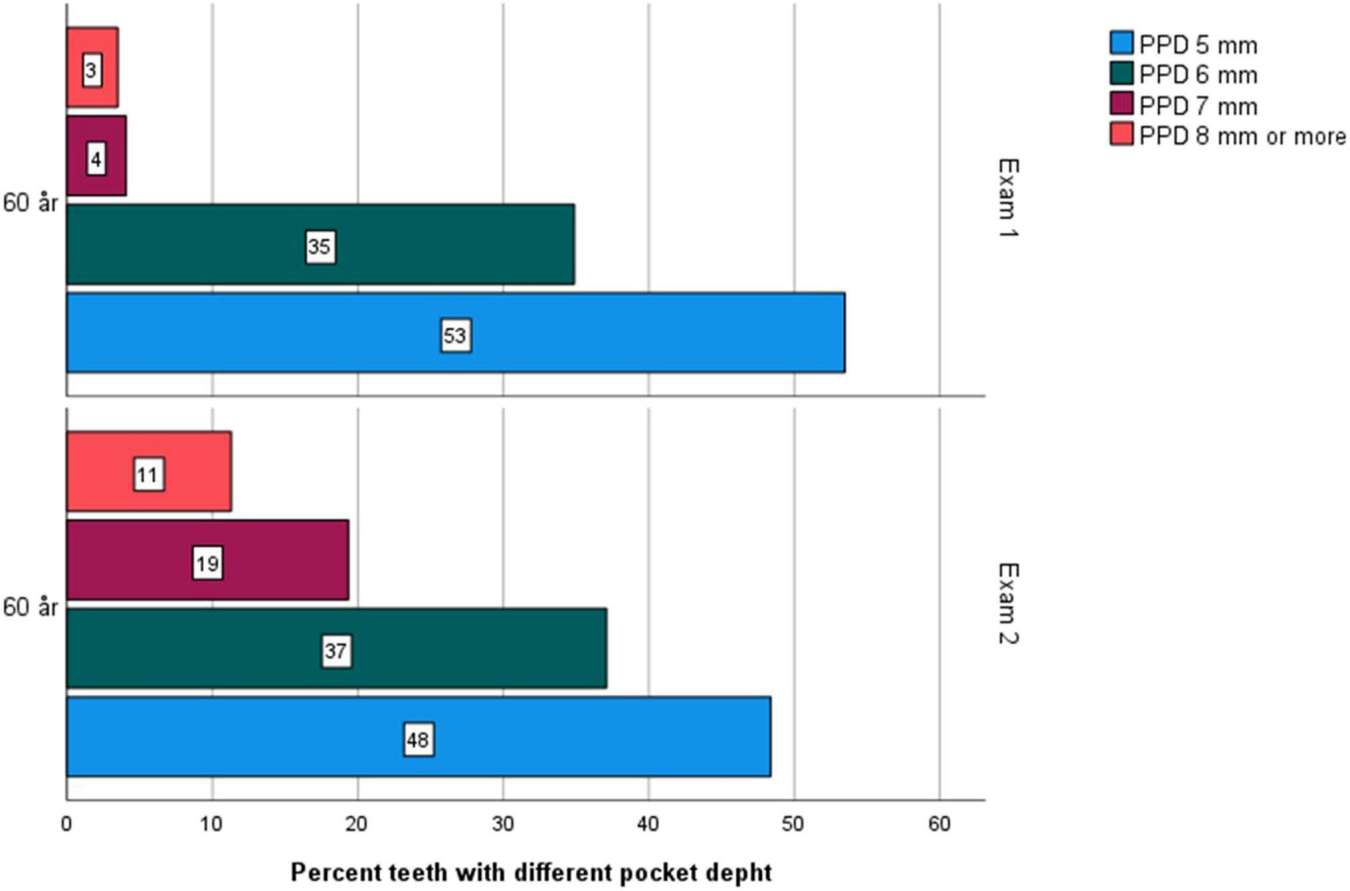
Illustration of percent teeth with different pocket depth 5 mm, 6 mm, 7 mm, and 8 mm or more in the age cohorts of 60 years

For the periodontal status, the presence of at least one pocket PPD ≥ 5 mm or PPD ≥ 6 mm, BOP, and one site or more with bone loss ≥ 5 mm showed no significant differences between the cohorts at the two different examination years (Table 3). The percentage of teeth with different PPD is presented in Fig. 2. Gingivitis was present in 65.2% of the individuals at exam 1, compared to 67.8% at exam 2 (NS). A diagnosis of periodontitis was found in 17.0% of the individuals at exam 1 compared to 15.9% at exam 2 (NS). Bacterial coatings (grades 2–4) decreased between exam 1 and exam 2 (*p* < 0.006) (Table 4).

**Table 3:** Clinical and radiographic presence of periodontal pocket depth (PPD) ≥ 5 mm, ≥ 6 mm, bleeding on probing (BOP), and bone level in dentate individuals of age cohorts 60 years and 81 years at exam and at exam 2

Variables	Exam 1 60 y *n* = 165	Exam 2 60 y *n* = 64	*p* value	Exam 1 81 y *n* = 101	Exam 2 81 y *n* = 48	*p* value
PPD ≥ 5 mm						
n/t (%)	105/165 (63.6)	35/62 (56.5)	NS	55/101 (54.5)	32/48 (66.7)	NS
PPD ≥ 6 mm						
n/t (%)	60/165 (36.4)	23/62 (37.1)	NS	21/101 (20.8)	19/48 (39.6)	0.016^§^
BOP						
Mean (SD)	21.71 (21.48)	19.11 (17.20)	NS	24.74 (25.29)	21.60 (26.70)	NS
Range (%)	0–100	0–100		0–100	0–100	
*n*	164	63		100	48	
Bone level						
≥ 5 mm						
n/t (%)	90/162 (55.6)	41/64 (64.0)	NS	74/100 (74.0)	43/48 (89.5)	0.029^§^

Notes: PPD was calculated on a tooth level; Presence of PPD ≥ 5 mm, ≥ 6 mm, and bone level ≥ 5 mm at least one site per individual. Presence of bone level was measured by x-ray at the mesial and distal surfaces. ^§^Statistical significance was determined at *p* < 0.05. For analysis of periodontal pocket depth and bone level Pearson’s χ^2^ test were used. For analysis of BOP, independent *t* test (equal variance not assumed) was used; *NS*, non-significant

**Table 4:** Frequency (%) of dentate individuals with bacterial coating based on visual grading from 1 to 4 by mucosal plaque index (MPI) in individuals 60 years and 81 years at exam 1 and at exam 2, respectively

Grading scale	Exam 1 60 y *n* = 162^1^	Exam 2 60 y *n* = 64	Exam 1 81 y *n* = 101	Exam 2 81 y *n* = 48
1. No soft coatings can be seen	61.6	79.7	40.0	68.8
2. Small amounts of barely visible coating	33.3	17.2	43.0	25.0
3. Moderate amounts of coatings	4.4	1.6	13.0	6.3
4. Ample amounts of cohesive coatings	0.6	1.6	4.0	0.0

Notes: ^1^Three missing cases

### Cohort groups: 81 years

The proportion of dentate individuals increased from 82.0% at exam 1 to 94.1% at exam 2 (*p* < 0.038). The mean number of teeth increased from 14.3 (SD ± 7.26) at exam 1 to 20.2 (SD ± 6.46) at exam 2 (*p* < 0.001) (Fig. 1), and presence of 20 teeth or more increased from 31.0% at exam 1 to 62.5% at exam 2 (*p* < 0.001). The proportion of individuals with removable prostheses decreased between the time intervals from 49.6% at exam 1 to 13.7% at exam 2 (*p* < 0.001). Dental implants were found in 11.6% at exam 1 and in 23.5% at exam 2 (*p* < 0.046). At exam 1, the mean value of dental implants was 5.43 (SD ± 2.56), and the corresponding figure at exam 2 was 5.83 (SD ± 3.04).

Prevalence of intact teeth, root residues, root fillings, manifest buccal/lingual caries, and approximal caries in all dentate individuals showed no significant differences between exam 1 and 2. In Table 2, the mean number of intact teeth, in the dentate individuals, root residues, root fillings, and manifest buccal/lingual and approximal caries is presented.

**Fig. 3:**
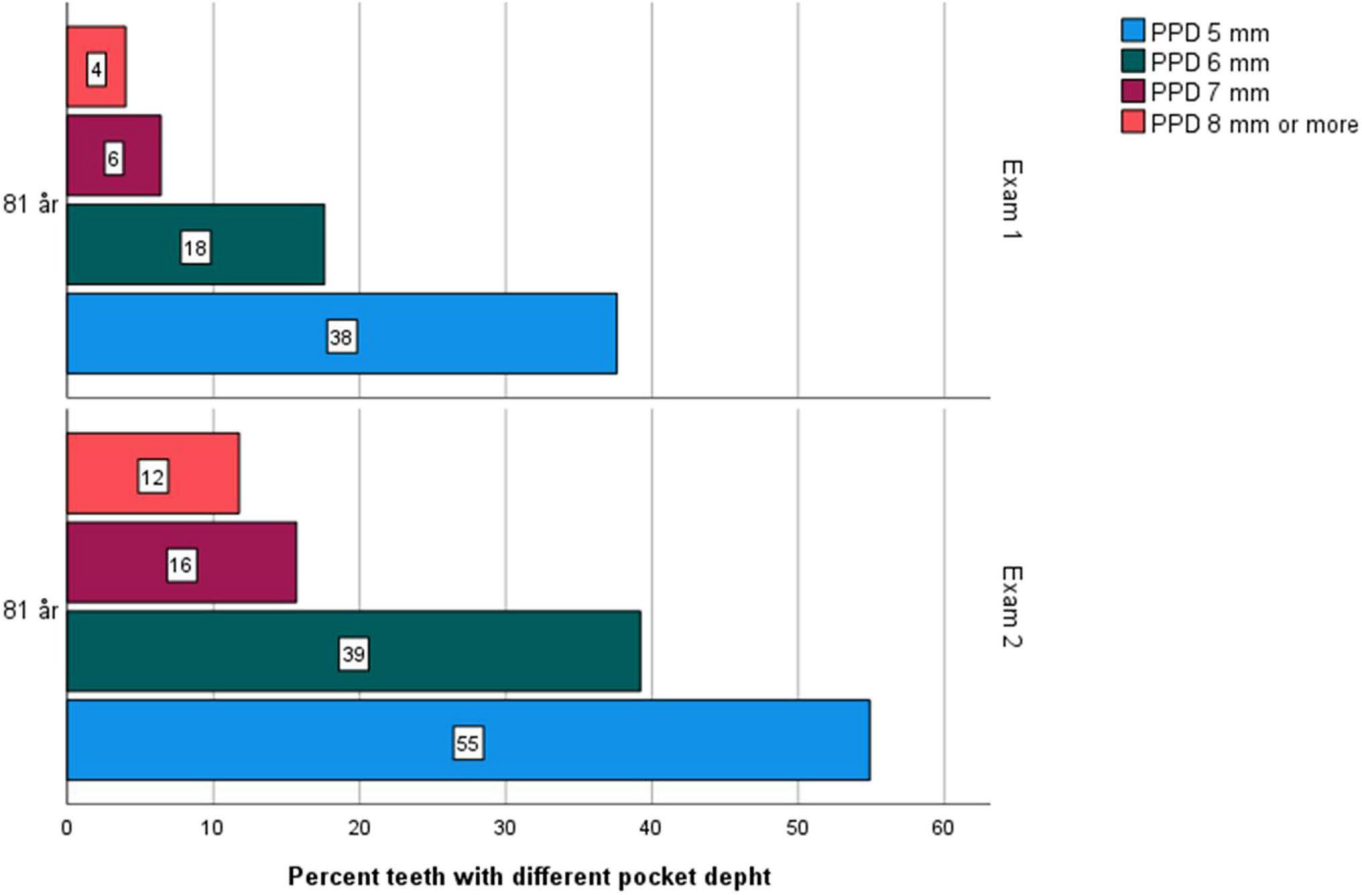
Illustration of percent teeth with different pocket depth 5 mm, 6 mm, 7 mm, and 8 mm or more in the age cohorts of 81 years

The presence of PPD ≥ 6 mm, and bone level ≥ 5 mm on at least one site per individual increased significantly from exam 1 to 2 (*p* < 0.016 and *p* < 0.029, respectively) (Table 3). The distribution of teeth with different PPD is presented in Fig. 3. Gingivitis was found in 61.4% of the individuals at exam 1 and in 52.1% at exam 2 (NS), and periodontitis was present in 21.8% at exam 1 compared to 31.2% at exam 2 (NS). The presence of bacterial coating (grades 2–4) decreased between exam 1 and exam 2 (*p* < 0.001) (Table 4).

## Discussion

The present study showed an improvement in the oral health status between exams 1 and 2 in both the 60- and 81-year-old age cohorts. Over the 13 years, the number of teeth increased. An increase in number of teeth over time agrees with other recent Swedish studies [35, 36]. However, in the present study, the mean of remaining teeth was higher than the findings in the UK [37] and in repeated population-based studies in oral health trends in the USA [5, 38].

In the present study at exam 2, most individuals in the 60-year-old cohort and more than half in the 81-year-old cohort had twenty or more teeth. This figure is higher than in studies from Finland [39] and the UK [40]. Based on data from the present study, one of WHO’s global oral health goals having twenty or more teeth [41] has already been attained. Having twenty or more teeth has been determined to be necessary to achieve a good masticatory ability [42, 43]. One possible explanation for the fact that older individuals in Sweden retain more natural teeth than in the USA and some European countries is the introduction of dental care insurance in 1974. This commitment contributed to the expansion of dental care and increased number of dental professions. In addition, improved utilization led to regular dental recall system and investment in preventive oral health care [44, 45] and has resulted in an improved oral health status among the population.

The majority of individuals in both cohort groups had no diagnosis of manifest caries. The mean number of manifest caries lesions did not significantly differ in the age group of 60 years or 81 years. Water fluoridation, implementation of fluoride toothpaste, fluoride mouth rinses for controlling caries during childhood [46], and greater access to dental care [45] might have benefitted both age cohorts. Regular visits to dental hygienists increased from 26% in 1992 to 57.2% in 2012 in Sweden especially in the older population [47]. We can assume that repeated oral health information and reinstructions in oral hygiene during dental hygienist’s visits may have contributed to positive attitudes toward preventive care and greater awareness in oral hygiene in both age cohorts in this study. However, in contrast to our study, Norderyd et al. [35] showed that the mean value of decayed (manifest and initial lesions) and filled teeth increased in the age groups 70 and 80 years for 40 years, while in the age group of 60 years, caries lesions decreased. Edman et al. [48] found decreased mean value between 1983 and 2013 of decayed surfaces in 65-year-olds and 75-year-olds but increased in 85-year-olds. The differences between these studies and ours can be that we used panoramic radiographs for caries registration which may have contributed to underestimating of approximal caries.

However, it is generally considered that older individuals retain more natural teeth nowadays, which was confirmed in the present study. It is worth noting that the most significant change in the present study was shown in the oldest group with nearly sex more retaining teeth, about twice as many as in the younger cohorts. The critical question is, what will happen when older people cannot manage their oral hygiene anymore and become dependent on help? Previously published data on dependent individuals 65 years and over showed that manifest caries was common. One in four individuals was at high risk for deterioration of general health or oral health [27]. In the USA (State BSS report and NHANES 2011–2016), the mean of untreated decayed teeth was higher among home-limited and care dependent individuals 65 years and older in comparison to independent individuals in the same age [49]. These two studies indicate that caries prevalence increases with care dependency and rising age.

Over the 13 years, no significant differences were found in the prevalence of gingivitis and periodontitis in any of the age cohorts. Nevertheless, among the 81-year-olds, there was a statistical increase in PPD and bone loss at exam 2. Differences in PPD and bone loss in the age cohorts 60 years were not observed. In contrast to the present study, Wahlin et al. [50] showed a non-significant decrease concerning the presence of at least one PPD ≥ 6 mm in individuals 80 years. A possible explanation for the increase of deeper PPD and bone loss in the older group in our study could be the increased number of remaining teeth. In the present study, majority of 81-year-old individuals had twenty teeth or more which may explain the changes mentioned above in periodontal status. The strength of our study is that measurements of PPD and bone level are performed in all permanent teeth, which reduce the risk for over and under estimation [51]. Panoramic radiographs were used for registration of bone level which is common in dentistry and has been reported to have good reliability compared to intraoral radiographs in assessing bone level [52].

However, differences in examination methods and definitions of periodontitis make it challenging to make comparisons. Our results of deteriorating periodontal status in the older age cohorts are a challenge for both the older individuals and the dental professionals when dependency becomes a reality. Schwendicke et al. [15] confirmed that periodontal treatment needs over time increased in older individuals 65 years and older. Knowledge about periodontal conditions in older individuals is a crucial element for preventive care to maintain a good oral health status for life.

With increasing age, health status can gradually or rapidly be changed both medically and socially, which can also affect oral health status [53]. These changes can lead to dependency, extensive dental care needs, and reduced quality of life. Dental staff can be faced with an ethical dilemma when it comes to decide what can be remedied or not [54]. Therefore, regular dental visits, including oral examination and periodontal treatment, are vital to accomplish, not least when dependence of help with daily life increases. This might have a fundamental value for health in general for older individuals. However, in order to increase the understanding of oral status changes over time and with increasing age, longitudinal follow-up studies are necessary before more general conclusions can be drawn.

## Conclusion

Over 13 years, oral health improved for both 60- and 81-year-old age groups. The most significant changes were in the 81-year-old where oral health improved except for the periodontal status.

## References

[1] United Nations (UN) (2017) Monitoring of population programmes, focusing on changing population age structures and sustainable development, in the context of the full implementation of the Programme of action of the International Conference on Population and Development: report of the Secretary-General. United Nations digital library. https://digitallibrary.un.org/record/859428. Accessed 1 March 2020

[2] World Health Organization (WHO) (2018) World oral health report: Aging and health. Global World Health Organization. https://www.who.int/news-room/fact-sheets/detail/ageing-and-health. Accessed 3 March 2020

[3] Statistics Sweden (SCB) (2021) The future population of Sweden 2021–2070. Demographic reports 2021:1. SCB Statistic Sweden. https://www.scb.se. Accessed 26 August 2021

[4] Niccoli T, Partridge L (2012). Ageing as a risk factor for disease. Curr Biol.

[5] Griffin SO, Jones JA, Brunson D, Griffin PM, Bailey WD (2012). Burden of oral disease among older adults and implications for public health priorities. Am J Public Health.

[6] Clark D, Kotronia E, Ramsay SE (2021). Frailty, aging, and periodontal disease: basic biologic considerations. Periodontol 2000.

[7] van der Putten G-J, Brand HS, Schols JMGA, de Baat C (2011). The diagnostic suitability of a xerostomia questionnaire and the association between xerostomia, hyposalivation and medication use in a group of nursing home residents. Clin Oral Investig.

[8] Villa A, Wolff A, Narayana N, Dawes C, Aframian DJ, Lynge Pedersen AM, Vissink A, Aliko A, Sia YW, Joshi RK, McGowan R, Jensen SB, Kerr AR, Ekström J, Proctor G (2016). World workshop on oral medicine VI: a systematic review of medication-induced salivary gland dysfunction. Oral Dis.

[9] Tan ECK, Lexomboon D, Sandborgh-Englund G, Haasum Y, Johnell K (2018). Medications that cause dry mouth as an adverse effect in older people: a systematic review and metaanalysis. J Am Geriatr Soc.

[10] Mese H, Matsuo R (2007). Salivary secretion, taste and hyposalivation. J Oral Rehabil.

[11] Petersen PE, Yamamoto T (2005). Improving the oral health of older people: the approach of the WHO global oral health programme. Community Dent Oral Epidemiol.

[12] Hajishengallis G, Lamont RJ (2021). Polymicrobial communities in periodontal disease: their quasi-organismal nature and dialogue with the host. Periodontol 2000.

[13] GrönbeckLindén I, Hägglin C, Gahnberg L, Andersson P (2017). Factors affecting older persons’ ability to manage oral hygiene: a qualitative study. JDR Clin Trans Res.

[14] Lopez R, Smith PC, Göstemeyer G, Schwendicke F (2017). Aging, dental caries and periodontal diseases. J Clin Periodontol.

[15] Schwendicke F, Krois J, Kocher T, Hoffman T, Micheelis W, Jordan RA (2018). More teeth in more elderly: periodontal treatment needs in Germany 1997–2030. J Clin Periodontol.

[16] Glick M, Monteiro da Silva O, Seeberger GK, Xu T, Pucca G, Williams DM, Kess S, Eisele J-L, Severin T (2012). FDI Vision 2020: shaping the future of oral health. Int Dent J.

[17] Kassebaum NJ, Smith AGC, Bernabé E, Fleming TD, Reynolds AE, Vos T, Murray CJL, Marcenes W, GBD 2015 Oral health collaborators (2017). Global, regional, and national prevalence, incidence, and disability adjusted life years for oral conditions for 195 countries, 1990–2015: A systematic analysis for the global burden of diseases, injuries, and risk factors. J Dent Res.

[18] Kassebaum NJ, Bernabé E, Dahiya M, Bhandari B, Murray CJ, Marcenes W (2014). Global burden of severe periodontitis in 1990–2010: a systematic review and meta-regression. J Dent Res.

[19] Marcenes W, Kassebaum NJ, Bernabé E, Flaxman A, Naghavi M, Lopez A, Murray CJL (2013). Global burden of oral conditions in 1990–2010: a systematic analysis. J Dent Res.

[20] Al-Nasser L (2000). Lamster IB (2020) Prevention and management of periodontal diseases and dental caries in the older adults. Periodontol.

[21] Chung S-Y, Song K-B, Lee SG, Choi Y-H (2011). The strength of age effect on tooth loss and periodontal condition in Korean elderly. Arch Gerontol Geriatr.

[22] Renvert S, Persson RE, Persson GR (2013). Tooth loss and periodontitis in older individuals: results from the Swedish national study on aging and care. J Periodontol.

[23] BengtssonWallin V, Persson GR, Berglund J, Renvert S (2016). A cross-sectional study of the associations between periodontitis and carotid arterial calcifications in an elderly population. Acta Odontol Scand.

[24] Masood M, Newton T, Bakri Nazahiah N, Khalid T, Masood Y (2017). The relationship between oral health and oral health related quality of life among elderly people in United Kingdom. J Dent.

[25] Lamster IB (2016). Geriatric periodontology: how the need to care for the aging population can influence the future of the dental profession. Periodontol 2000.

[26] Lamster IB, Asadourian L, Del Carmen T, Friedman PK (2016). The aging mouth: differentiating normal aging from disease. Periodontol 2000.

[27] Andersson P, Renvert S, Sjögren P, Zimmerman M (2017). Dental status in nursing home residents with domiciliary dental care in Sweden. Community Dent Health.

[28] BengtssonWallin V, Persson GR, Berglund J, Renvert S (2019). Carotid calcification in panoramic radiographs are associated with future stroke or ischemic heart diseases: a long-term follow-up study. Clin Oral Investig.

[29] Curtis DA, Lin GH, Rajendran Y, Gessese T, Suryadevara J, Kapila YL (2021). Treatment planning considerations in the older adult with periodontal disease. Periodontol 2000.

[30] Jin L, van Dijk W (2014). Reinforcing and refining oral healthcare. Int Dent J.

[31] Lagergren M, Fratiglioni L, Hallberg Rahm I, Berglund J, Elmståhl S, Hagberg B, Holst G, Rennemark M, Sjölund B-M, Thorslund M, Wiberg I, Winblad B, Wimo A (2004). A longitudinal study integrating population, care and social services data. The Swedish National study on Aging and Care (SNAC). Aging Clin Exp Res.

[32] Fagerström C, Palmqvist R, Carlsson J, Hellström Y (2011). Malnutrition and cognitive impairment among people 60 years of age and above living in regular housing and in special housing in Sweden: a population-based cohort study. Int J Nurs Stud.

[33] World Medical Association (WMA). Declaration of Helsinki (2013) Ethical principles for medical research involving human subjects, adopted by the 18th WMA General Assembly, Helsinki, Finland, June 1964, latest revision by the WMA General Assembly, Seoul 2013. Ferney-Voltaire, France

[34] Henriksen BM, Ambjørnsen E, Axéll TE (1999). Evaluation of a mucosal-plaque index (MPS) designed to assess oral care in groups of elderly. Spec Care Dentist.

[35] Norderyd O, Koch G, Papias A, Kohler AA, Helkimo AN, Brahm CO, Lindmark U, Lindfors N, Mattsson A, Rolander B, Ullbro C, Gerdin EW, Frisk F (2015). Oral health of individuals aged 3–80 in Jönköping, Sweden, during 40 years (1973–2013): II. Review of clinical and radiographic findings. Swed Dent J.

[36] Edman K, Öhrn K, Holmlund A, Nordström B, Hedin M, Hellber D (2012). Comparison of oral status in an adult population 35–75 year of age in the county of Dalarna, Sweden in 1983 and 2008. Swed Dent J.

[37] Steele JG, Treasure ET, O´Sullivan I, Moris J, Murray J (2012). Adult dental health survey 2009: transformations in British oral health 1968–2009. Br Dent J.

[38] Dye BA, Tan S, Smith V, Lewis BG, Barker LK, Thornton-Evans G, Eke PI, Beltrán-Aguilar ED, Horowitz AM, Li C-H (2007) Trends in oral health status: United States, 1988–1994 and 1999–2004. National center for health statistics. Vital Health Stat 11:248 17633507

[39] Suominen AL, Varsio S, Helminen S, Nordblad A, Lahti S, Knuutila M (2018). Dental and periodontal health in Finish adults in 2000 and 2011. Acta Odontol Scand.

[40] Fuller E, Steele J, Watt R, Nuttall N (2011) Oral health and function - a report from the Adult Dental Health Survey 2009. NHS Digital. http://www.hscic.gov.uk/catalogue/PUB01086/adul-dentheal-surv-summ-them-the1-2009-rep3.pdf. Accessed 14 December 2021

[41] World Health Organization (WHO) (1982). Global goals for oral health in the year 2000. Int Dent J.

[42] Moriya S, Tei K, Murata A, Muramatsu M, Inoue N, Miura H (2012). Perceived chewing ability and need for long-term care in the elderly: a 5-year follow-up study. J Oral Rehabil.

[43] Gotfredsen K, Walls AWG (2007). What dentition assures oral function?. Clin Oral Implants Res.

[44] The National Board of Health and Welfare (2019) Tillståndet och utvecklingen inom hälso- och sjukvård och tandvård. Lägesrapport. Socialstyrelsen https://www.socialstyrelsen.se/globalassets/sharepoint-dokument/artikelkatalog/ovrigt/2019-3-2.pdf. In Swedish. Accessed 1 February 2020

[45] Ahacic K, Thorslund M (2008). Changes in dental status and dental care utilization in the Swedish population over three decades: age, period, or cohort effects?. Community Dent and Oral Epidemiol.

[46] Thorell P, Ericsson Y (1965). Two-year clinical tests with different methods of local caries-preventive fluorine application in Swedish school-children. Acta Odontol Scand.

[47] Åström AN, Ekbäck G, Ordell S, Lie SA, Gulcan F (2017). Dental hygienist attendance and its covariates in an ageing Swedish cohort. Eur J Oral Sci.

[48] Edman K, Öhrn K, Nordström B, Holmlund A (2016). Prevalence of dental caries and influencing factors, time trends over a 30-year period in an adult population. Epidemiological studies between 1983 and 2013 in the county of Dalarna. Sweden. Acta Odontol Scand.

[49] Griffin SO, Griffin PM, Li C-H, Bailey WD, Brunson D, Jones JA (2019). Changes in older adults’ oral health and disparities: 1999–2004 and 2011–2016. J Am Geriatr Soc.

[50] Wahlin Å, Papias A, Jansson H, Norderyd O (2018). Secular trends over 40 years of periodontal health and disease in individuals aged 20–80 years in Jönköping, Sweden: repeated cross-sectional studies. J Clin Periodontol.

[51] Holtfreter B, Albandar JM, Dietrich T, Dye BA, Eaton KA, Eke PI, Papapanaou PN, Kocher T (2015). Standards for reporting chronic periodontitis prevalence and severity in epidemiologic studies: proposed standards from the joint EU/USA periodontal epidemiology working group. J Clin Periodontol.

[52] Persson RE, Tzannetou S, Feloutzis AG, Brägger U, Persson GR, Lang NP (2003). Comparison between panoramic and intra-oral radiographs for the assessment of alveolar bone levels in a periodontal maintenance population. J Clin Periodontol.

[53] Finbarr A (2019). Pragmatic care for an aging compromised dentition. Aust Dent J.

[54] Alsaleh A, Kapila A, Shahriar I, Kapila YL (2021). Dental informed consent challenges and considerations for cognitively impaired patients. Periodontol 2000.

